# Sensitivity of global storm surge modelling to sea surface drag

**DOI:** 10.1007/s10236-025-01713-3

**Published:** 2025-07-19

**Authors:** Feyza Nur Özkan, Martin Verlaan, Sanne Muis, Firmijn Zijl

**Affiliations:** 1 Delft Institute of Applied Mathematics, Delft University of Technology, Mekelweg 4, 2628 CD Delft, The Netherlands; 2 Hydrodynamics and Forecasting, Deltares, P.O. Box 177, 2600 MH Delft, The Netherlands; 3 Institute for Environmental Studies, Vrije Universiteit Amsterdam, De Boelelaan 1111, 1081 HV Amsterdam, The Netherlands

**Keywords:** Extreme events, Storm surge, Wind stress, Atmosphere-ocean coupling, Global hydrodynamic model

## Abstract

Accurate storm surge modeling is essential for predicting coastal flooding and mitigating impacts on vulnerable regions. This study evaluates the influence of different sea surface drag parameterizations on surge predictions using the Global Tide and Surge Model (GTSM) over a 10-year period (2006–2015) and two storm events. Four model experiments were tested, ranging from a fully dynamic formulation, including variable air density, atmospheric stability, and sea-state-dependent drag, to a simplified constant-drag approach. Results show that advanced drag formulations reduced the underestimation of annual maximum surge values from 18% to 12% globally, with the variable Charnock parameter contributing the most. Conversely, using a constant Charnock value and thereby neglecting wave-dependent roughness increases prediction errors, especially in regions with highly variable sea states. Case studies of Storm Xaver (2013) and Hurricane Fiona (2022) show that advanced parameterizations better capture wind stress variations, reducing root mean square error from 0.21 m to 0.16 m for Xaver and improving surge predictions by up to 0.30 m for Fiona. Consistent with earlier studies, a persistent underestimation of extreme surge events remains across all experiments. While wave-dependent roughness improves performance, no single parameter fully explains this bias. However, wave-dependent roughness particularly enhances model performance in high-latitude and storm-prone areas, where sea state and atmospheric conditions vary widely. Our results show that variations in air density and atmospheric stability have minimal impact on surge height. As such, prioritizing the implementation of dynamic, sea-state-dependent drag formulations, particularly variable Charnock, is key to further improving the accuracy of storm surge forecasting systems and future projections.

## Introduction

Storm surges are potentially devastating elevations in sea level caused by both extra-tropical and tropical systems. They can significantly impact coastal regions, inducing flooding and erosion that disrupt social, economic, and environmental dynamics (Resio and Westerink 2008). The most devastating impact of tropical cyclones is often due to coastal flooding induced by storm surges (McInnes et al. 2003). Hurricane Katrina in 2005 resulted in extensive flooding in New Orleans, leading to approximately 800 fatalities and approximately 40 billion in damages (Jonkman et al. 2009; Nicholls et al. 2015). Similarly, Typhoon Haiyan in Philippines led to the death or disappearance of around 8,000 people and the destruction of one million homes, primarily due to extreme sea levels (LeComte 2014). Extra-tropical storms can also generate significant surges that lead to severe impacts, as occurred in the North Sea flood in 2013 (Spencer et al. 2015) and Cyclone Xynthia in 2010 in France (Chadenas et al. 2014).

Storm surges result from a storm’s low atmospheric pressure and high surface winds. The effect of atmospheric pressure is represented by the inverse barometric effect, which quantifies how sea level rises as atmospheric pressure falls (Pugh 2011). In addition, storm surface winds interact with the ocean surface, generating wind stress that drives water movement and contributes to storm surge development. The characteristics of storm surges are influenced by the storm’s features such as intensity, direction, and size, but also by the coastal geometry and bathymetry (Bode and Hardy 1997; Powell and Reinhold 2007; Resio and Westerink 2008). The effect of wind on storm surges depends on water depth, with winds having a greater impact in shallow waters and diminishing influence in deeper areas. In contrast, the effect of atmospheric pressure is relatively uniform, with water depth playing a minor role (Flather 2000). Notably, coastal geometry can funnel water to higher levels. Thus, storm surges are typically higher in areas with shallow, wide continental shelves compared to areas with deep water and steep slopes.

Hydrodynamic models have become essential tools for modelling storm surges, with early applications primarily focusing on local and regional scales (e.g. Jelesnianski et al. 1992; Kim et al. 2008, 2010; Pineau-Guillou et al. 2014; Zijl et al. 2013). More recently, advancements in computational resources and the availability of global datasets have enabled the extension of these models to global scales (e.g. Kodaira et al. 2016; Verlaan et al. 2015; Pringle et al. 2021). Both regional and global storm surge models have been employed for forecasting, hindcasting, and projecting future water levels (e.g. Bode and Hardy 1997; Kleermaeker et al. 2017; Flather 2000; Lin et al. 2019; Muis et al. 2020; Vousdoukas et al. 2017), thereby informing efforts to improve disaster preparedness, flood protection, and climate adaptation.

**Fig. 1:**
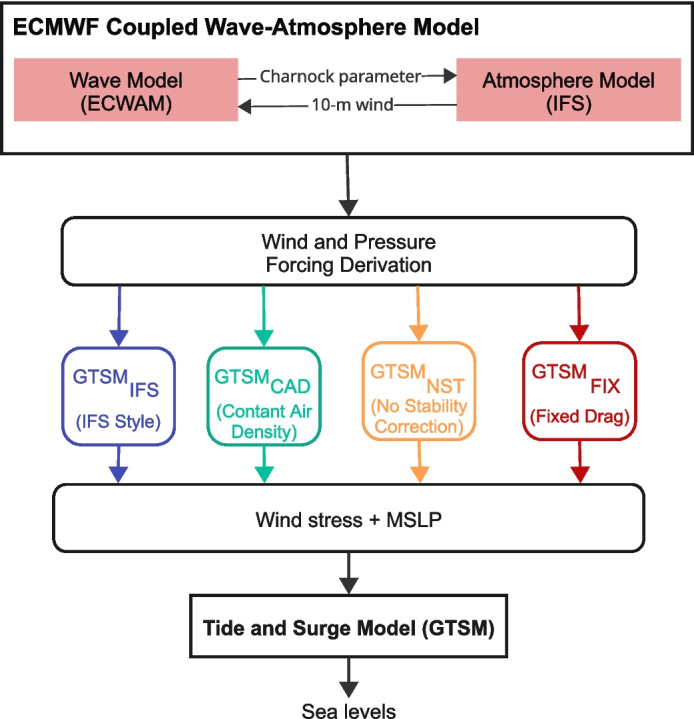
Flowchart of the modelling approach that is used to simulate global storm surges using different four parametrization for wind stress

Charnock’s formulation has been widely applied in both global-scale (e.g., Madec and NEMO System Team 2023; Verlaan et al. 2015; Westerink et al. 2008) and regional storm surge models (e.g., Kim et al. 2008, 2010, 2015; Pineau-Guillou et al. 2014; Muller et al. 2014; Zijl et al. 2013). While simplified approaches using a constant Charnock parameter remain common (Bresson et al. 2018; Brown et al. 2013; Dullaart et al. 2020; Kim et al. 2015; Muis et al. 2016, 2019; Vatvani 102334 2012), a variable Charnock parameter has long been the state-of-the-art (Mastenbroek et al. 1993; Nicolle et al. 2009; Muller et al. 2014; Bertin et al. 2015; Pineau-Guillou et al. 2020). The role of wave-dependent surface stress in storm surge modeling has been extensively studied, Mastenbroek et al. (1993) demonstrated that young, steep waves amplify surface drag, significantly altering surge simulations in the North Sea. Additionally, Oost et al. (2002) showed that wave age is a key factor in modulating the drag coefficient through sea state conditions. In shallow coastal areas, Nicolle et al. (2009) found that wave-tide-surge interactions can increase storm surges due to higher sea surface drag and bottom stress, highlighting the need to include wave effects in regional models. Bertin et al. (2015) showed that wave radiation stress and young waves contributed to Xynthia’s surge, while Muller et al. (2014) found that wave-dependent Charnock formulations improve storm surge accuracy, especially with high-resolution meteorological data along the French Atlantic and English Channel coasts. Pineau-Guillou et al. (2018) showed that traditional wave-age-dependent parameterizations can produce unrealistic drag values and proposed an adjusted Charnock formulation to improve wind stress and surge predictions. Building on this, Pineau-Guillou et al. (2020) showed that using a wave-dependent stress formulation improved surge simulations, especially under young and rough sea conditions. Despite extensive research on wave-dependent wind stress formulations, key factors like atmospheric stability and air density variations are underrepresented in storm surge modelling. Studies such as Rastigejev et al. (2011), Andreas et al. (2015), and Shabani et al. (2014) demonstrate that stratification in the marine boundary layer can affect surface fluxes and drag coefficients, particularly under varying stability conditions. Notably, Kara et al. (2005) showed that stability has an influence on the drag coefficient, especially at low to moderate wind speeds, yet stability effects are rarely included in surge models. Similarly, air density is often assumed constant e.g., (Dullaart et al. 2020; Muis et al. 2019; Muller et al. 2014; Ridder et al. 2018), leading to a fixed relationship between wind speed, roughness length, and, in turn, stress. These factors, though important for air-sea momentum transfer, remain largely unstudied in the context of storm surge modeling.

The goal of this study is to provide a global assessment of the impact of different aspects of sea surface drag parameterization on storm surge modeling. While many regional studies have explored individual drag-related factors, comprehensive global evaluations particularly those considering the role of air density and atmospheric stability remain limited. Using the Global Tide and Surge Model (GTSM) (Wang et al. 2022), which previously relied on a constant Charnock parameter for wind stress representation (Dullaart et al. 2020, 2021; Muis et al. 2016, 2019, 2020), we introduce a more physically realistic approach that better captures ocean-atmosphere interactions and atmospheric variability. The new parameterizations are aligned with those implemented in the Integrated Forecasting System (IFS) (ECMWF 2023a), ensuring consistency with state-of-the-art atmospheric and oceanographic modeling. To systematically evaluate the sensitivity to different drag formulations, we conduct four modeling experiments covering the period 2006–2015, progressively simplifying the representation of air–sea momentum transfer. These include: 1) a fully dynamic approach with a sea-state-dependent Charnock parameter and wind fields adjusted for variable air density and atmospheric stratification; 2) a sea-state-dependent Charnock parameter with neutral wind fields, neglecting air density variations; 3) a sea-state-dependent Charnock parameter with actual (non-neutral) winds, neglecting air density and stratification corrections; and 4) a fixed Charnock parameter with actual winds, neglecting sea-state dependence, air density variations, and atmospheric stability corrections, representing the simplest formulation. By comparing modeled surge heights with observations, we assess the relative importance of sea state, air density, and atmospheric stability in drag parameterizations and their influence on model performance across the globe. This study aims to determine whether incorporating more complex physical processes significantly improves storm surge predictions or if a simpler approach remains sufficient.

The paper is organized such that the next sections first present our data and methodology, followed by an in-depth analysis of the results that informs sensitivity and improvements of storm surge modeling. Specifically, Section 2 introduces the data and methodology, including a description of the employed storm surge model, GTSM, reanalysis data for the forcing field, observational data for validation of the model experiments, and the conducted model experiments. Section 3 then presents the study’s findings by comparing the different model experiments’ performance against observations and the default experiment of GTSM. Section 4 evaluates the study’s findings and provides concluding remarks, summarizing the main outcomes.

## Data and methods

Our approach uses wind-drag parameterizations derived from ERA5 reanalysis data, providing spatially and temporally varying forcing grids for GTSM. We assess model performance over a 10-year period (2006–2015) to capture robust statistics, while also conducting detailed analyses of two individual storm events (i.e., Xaver in 2013 and Fiona in 2022). Four wind-drag experiments are evaluated, ranging from the most advanced to the most simplified representations of air-sea momentum transfer. These include the IFS-style Drag Experiment ($\text {GTSM}_{\text {IFS}}$), Constant Air Density Experiment ($\text {GTSM}_{\text {CAD}}$), No Stability Correction Experiment ($\text {GTSM}_{\text {NST}}$), and Fixed Drag Experiment ($\text {GTSM}_{\text {FIX}}$). These experiments are compared against observational data from tide gauge records to quantify their impact on the accuracy of storm surge simulations. The data processing workflow is summarized in Fig. 1.

### ERA5 reanalysis data

The forcing fields for GTSM are derived from ERA5 reanalysis dataset, as provided by the European Centre for Medium-Range Weather Forecasts (ECMWF; Hersbach et al. (2020)). This study uses the parameters listed in Table 1, which have a $0.25^\circ \times 0.25^\circ$ horizontal resolution (approximately 31-km spatial grid spacing) and cover 2006-2015 with an hourly interval. These parameters originate from a four-dimensional variational data assimilation and model forecasting system of the Integrated Forecasting System. The IFS’s atmospheric model is coupled with both a land-surface model (HTESSEL) and an ocean-wave model (WAM). The ocean-wave model is based on the theoretical frameworks proposed by Janssen (1991) and Abdalla and Bidlot (2002), particularly addressing the effects of wave age, air density and gustiness on wave growth (ECMWF 2023b). The coupling of these models enables a more realistic representation of dynamic interactions and momentum exchange, where the atmosphere influences wave formation through surface wind stress, and in turn, the waves modify the atmospheric boundary layer by altering the surface roughness depending on the sea state. Within this framework, the characteristic length scale for momentum exchange is determined by a Charnock relation that varies with sea state, which connects the surface aerodynamic roughness length to the surface stress. This approach provides a time- and space-dependent Charnock parameter back to the atmosphere, as documented in ERA5 dataset. Further information regarding ERA5 reanalysis dataset and the ocean-wave model of IFS can be found in documents (ECMWF 2023b; Hersbach et al. 2020).

**Table 1:** The parameters used from ERA5 dataset

Variable	Acronym
Mean sea level pressure	msl
10 m u- and v- wind component	u10, v10
Neutral wind at 10 m u- and v- component	u10n, v10n
Charnock parameter	chnk
2 m temperature	2t
2 m dewpoint temperature	2d

### Overview of wind stress parameterization

The key to storm surge modeling is accurately representing air-sea momentum transfer, which is expressed through wind stress ($\tau$) in hydrodynamic models:

1 $$\begin{aligned} \tau = \rho _a C_D (U_{10})^2 \end{aligned}$$

where $\rho _a$ is the atmospheric density, $C_D$ is the drag coefficient, and $U_{10}$ is the wind speed at 10 meters above the surface (Pugh 2011). The drag coefficient, influenced by sea surface roughness and wind forcing, plays a role in storm surge dynamics. Under neutral atmospheric conditions, it is commonly expressed as:

2 $$\begin{aligned} C_D = \left( \frac{u_*}{U_{10}}\right) ^2 = \frac{\mathcal {K}^2}{\ln ^2\left( \frac{z}{z_0}\right) }, \end{aligned}$$

where $\mathcal {K}$ is the Von Kármán constant (0.40), *z* is the height above the surface (10 m), $u_*$ is the friction velocity, and $z_0$ is the aerodynamic roughness length. At low wind speeds, roughness is controlled by viscosity, while at higher speeds, it is largely governed by wave-related drag. The roughness length of the sea surface varies with the sea state and is typically parameterized as:

3 $$\begin{aligned} z_0 = z_{\text {0visc}} + z_{\text {0wave}} = \frac{0.11 \nu }{u_*} + \alpha _{\text {Ch}} \frac{u_*^2}{g}, \end{aligned}$$

where $\nu$ is the kinematic viscosity, *g* the gravitational acceleration, and $\alpha _{\text {Ch}}$ the Charnock parameter. The second term in Eq. 3, introduced by Charnock (1955), accounts for the influence of sea state on roughness length. The Charnock parameter ($\alpha _{\text {Ch}}$), which links surface roughness to wind stress, is often treated as constant but has been shown to vary with sea state, particularly wave age (Oost et al. 2002). Young, developing waves absorb more momentum, enhancing surface drag. Janssen (1991) parametrized the effect of wave growth on wind stress by modifying the wind profile, leading to an enhanced surface roughness length expressed through a wave-dependent Charnock parameter, as a function of the wave-induced stress ($\tau _w$). In this study, sea-state effects are dynamically represented following the ERA5 implementation of Janssen (1991)’s formulation, where the Charnock parameter is calculated as:

4 $$\begin{aligned} \alpha _{\text {Chvar}} = \frac{\hat{\alpha }}{\sqrt{1 - \tau _w / \tau }}, \quad \hat{\alpha } = 0.006, \end{aligned}$$

where $\tau _w$ represents the wave-induced stress. Because calculating $\tau _w$ depends on the wave spectrum, the ECMWF uses a coupled atmosphere-wave model within its IFS to estimate $\alpha _{\text {Chvar}}$ dynamically (Janssen 1991). This setup lets surface roughness and wind stress adjust as wave conditions change, helping to better capture how momentum is exchanged between the ocean and atmosphere.

The relationship between wind stress and the 10m wind is influenced by several factors, particularly the stability of the surface layer. According to Monin-Obukhov similarity theory (Monin and Obukhov 1954), the wind profile can be expressed as:

5 $$\begin{aligned} U(z) = \frac{u_*}{\mathcal {K}} \left[ \ln \left( \frac{z}{z_0}\right) - \Psi _m\left( \frac{z}{L}\right) \right] , \end{aligned}$$

where $u_*$ is the friction velocity and *k* is the von Kármán constant. The function $\Psi _m$ is the stability correction, which depends on atmospheric stratification: $\Psi _m > 0$ for stable conditions, $\Psi _m < 0$ for unstable conditions, and $\Psi _m = 0$ for neutral conditions, reducing the equation to the standard logarithmic wind profile.

Accurate wind speed measurements are crucial for estimating surface heat and momentum fluxes, such as wind stress, across the global ocean (Kara et al. 2008). Since wind stress depends on the square of wind speed (Eq. 1) and is influenced by local stratification, variations in stratification can lead to corresponding fluctuations in wind stress. To address this, the concept of neutral wind ($U_{10N}$) could be useful. Neutral wind represents the 10-m wind derived from surface stress, assuming a neutrally stratified boundary layer. It is estimated by converting actual (non-neutral) 10-m wind to surface stress, accounting for stability, and then back to wind while neglecting stability effects. This adjustment removes variability due to local stability conditions, simplifying wind stress calculations without needing explicit surface stress information while still retaining stratification effects (Hersbach 2008). Neutral wind can remove stability-related biases caused by sea-air temperature differences, which influence near-surface wind speeds. In stable conditions (cooler water, warmer air), turbulence decreases, leading to lower neutral winds than actual winds. In unstable conditions (warmer water, cooler air), turbulence increases, raising neutral winds above actual values.

Beyond simplifying wind stress calculations, using neutral wind can also serve as an alternative to explicitly applying the stability function in wind forcing models. Instead of correcting for stability effects through the stability function, models can be forced directly with neutral winds. This approach, as used in this study, allows for a consistent wind forcing dataset while implicitly accounting for stability influences, offering an alternative to traditional stability corrections. At ECMWF, scatterometer data is assimilated as equivalent-neutral 10-m vector wind in the four-dimensional variational data assimilation (4D-Var) component of the IFS. Because scatterometer data is thought to be more closely linked to surface stress than wind, using an observation operator that responds to neutral rather than non-neutral wind is expected to improve accuracy (Hersbach 2010).

In addition to stability effects, variations in air density can also influence wind stress, as wind stress is directly proportional to air density (Eq. 1). In storm surge modeling, air density is often assumed to be constant in time and space, but this simplification does not account for variations, especially in regions with large temperature gradients. Higher air density leads to stronger wind stress for a given wind speed. To account for this, neutral wind can be converted into stress-equivalent wind, which adjusts for local air density conditions, providing a more accurate representation of air-sea momentum transfer (de Kloe et al. 2017).

By using neutral winds, sea-state-dependent Charnock values, and local air density adjustments, we create wind stress fields that better capture the changing conditions at the ocean-atmosphere interface. These more realistic stress fields are then used to drive the GTSM in our model experiments.

### Global Tide and Surge Model (GTSM)

In this study, we use the Global Tide and Surge Model Version 4.1 (GTSMv4.1; Wang et al. (2022)). GTSM is a depth-averaged hydrodynamic model developed using the Delft3D Flexible Mesh framework (Kernkamp et al. 2011). GTSM has global coverage and utilizes an unstructured grid that varies spatially in resolution. The model is coarser in the deep ocean at approximately 25 km, but becomes finer, about 2.5 km, near global coastal areas and 1.25 km in European coast. In order to simulate storm surges, mean sea level pressure and wind components at 10 m are used as forcing fields. Previous research has demonstrated that GTSM generally shows a good agreement with observed water levels (Dullaart et al. 2020; Muis et al. 2016, 2019, 2020). However, it is worth noting that the model’s performance can be adversely affected by complex local conditions, such as insufficient resolution in certain areas, and is highly dependent on the quality of the meteorological forcing.

The focus of this research is on four model experiments designed to analyze the impact of different sea surface drag parameterizations on storm surge simulations, highlighting how variations in drag coefficient influence modelling accuracy. The evaluation of model performance covers the period from 1 January 2006 until 31 December 2015, focusing on two specific storm events to assess the model’s representation of surge behavior and dynamics under storm conditions. We generate yearly time series of total water levels–integrating both tide and surge to accurately capture tide-surge interactions–for each experiment. These experiments consist of a spin-up period of 15 days and a provide output at a 10 minutes across 43,734 output locations that have a varying resolution (Muis et al. 2020). To isolate surges from the total water levels, we remove the tidal component using hatyan software (Veenstra 2022), following the same approach used with the observational data (see Section 2.5).

### Model experiments

In this study, we conduct four model experiments to perform a sensitivity analysis on the effects of different sea surface drag parameterizations on simulated storm surges. The performance of hydrodynamic models is highly dependent on sea surface drag, and these experiments help to understand how various approaches to calculating the drag coefficient at the atmosphere-ocean interface can influence storm surge modelling. An overview of the parameterization differences between the model experiments is presented in Table 2. The experiments are described as follows:

1. $\text {GTSM}_{\text {IFS}}$ (IFS-style Drag Experiment): This experiment closely follows the wind stress formulation used in the IFS (ECMWF 2023a), incorporating spatial and temporal variations in air density, atmospheric stability, and sea-state-dependent roughness. In contrast to previous GTSM studies (Dullaart et al. 2020; Muis et al. 2016, 2019, 2020), which assumed constant air density (1.20 kg/m$^3$) and a fixed Charnock parameter (0.041), this setup applies physically consistent corrections to better represent air-sea momentum exchange. Forcing fields include mean sea level pressure, a variable Charnock parameter ($\alpha _{\text {Chvar}}$), and wind fields corrected for both air density and atmospheric stratification, referred to as stress-equivalent winds ($U_{10S}$) (see Appendix). The resulting wind stress is calculated as: $\tau = \rho _a C_D(U_{10S}, \alpha _{\text {Chvar}}) U_{10S}^2$.
2. $\text {GTSM}_{\text {CAD}}$ (Constant Air Density Experiment): This experiment isolates the effect of air density by replacing the spatially and temporally varying air density from $\text {GTSM}_{\text {IFS}}$ with a constant average value of 1.20 kg/m$^3$. Atmospheric stability is still accounted for using neutral winds ($U_{10N}$), and the Charnock parameter remains sea-state dependent ($\alpha _{\text {Chvar}}$). Forcing fields also include mean sea level pressure. Wind stress is calculated as: $\tau = \rho _a C_D(U_{10N}, \alpha _{\text {Chvar}}) U_{10N}^2$.
3. $\text {GTSM}_{\text {NST}}$ (No Stability Correction Experiment): This experiment evaluates the impact of atmospheric stability by replacing neutral winds ($U_{10N}$) with actual 10-m wind speeds ($U_{10}$), without applying any correction for atmospheric stratification. In doing so, we assume neutral atmospheric conditions and neglect explicit stability effects in the drag formulation. Air density is fixed at 1.20 kg/m$^3$, and the Charnock parameter remains sea-state dependent ($\alpha _{\text {Chvar}}$). Wind stress is computed as: $\tau = \rho _a C_D(U_{10}, \alpha _{\text {Chvar}}) U_{10}^2$.
4. $\text {GTSM}_{\text {FIX}}$ (Fixed Drag Experiment): This experiment evaluates the impact of using a fully simplified drag formulation. It follows the default GTSM settings by applying a constant Charnock parameter ($\alpha _{\text {Ch}} = 0.041$) and a fixed air density of 1.20 kg/m$^3$. In line with earlier studies (e.g., (Dullaart et al. 2020; Muis et al. 2016)), this relatively high Charnock value is considered representative of storm conditions. Actual 10-m wind field ($U_{10}$) are used without corrections for atmospheric stability, assuming neutral conditions. The drag coefficient remains fixed, with no adjustments for sea state, air density, or stability. This represents the most simplified wind stress parameterization, calculated as: $\tau = \rho _a C_D(U_{10}, \alpha _{\text {Ch}}) U_{10}^2$.

**Table 2:** Overview of model experiments

Experiment	Air Density ($\rho _a$)	Wind	Charnock
$\text {GTSM}_{\text {IFS}}$	Variable	$U_{10S}$	$\alpha _{\text {Chvar}}$
$\text {GTSM}_{\text {CAD}}$	$1.20~\text {kg/m}^3$	$U_{10N}$	$\alpha _{\text {Chvar}}$
$\text {GTSM}_{\text {NST}}$	$1.20~\text {kg/m}^3$	$U_{10}$	$\alpha _{\text {Chvar}}$
$\text {GTSM}_{\text {FIX}}$	$1.20~\text {kg/m}^3$	$U_{10}$	$\alpha _{\text {Ch}}=0.041$

**Fig. 2:**
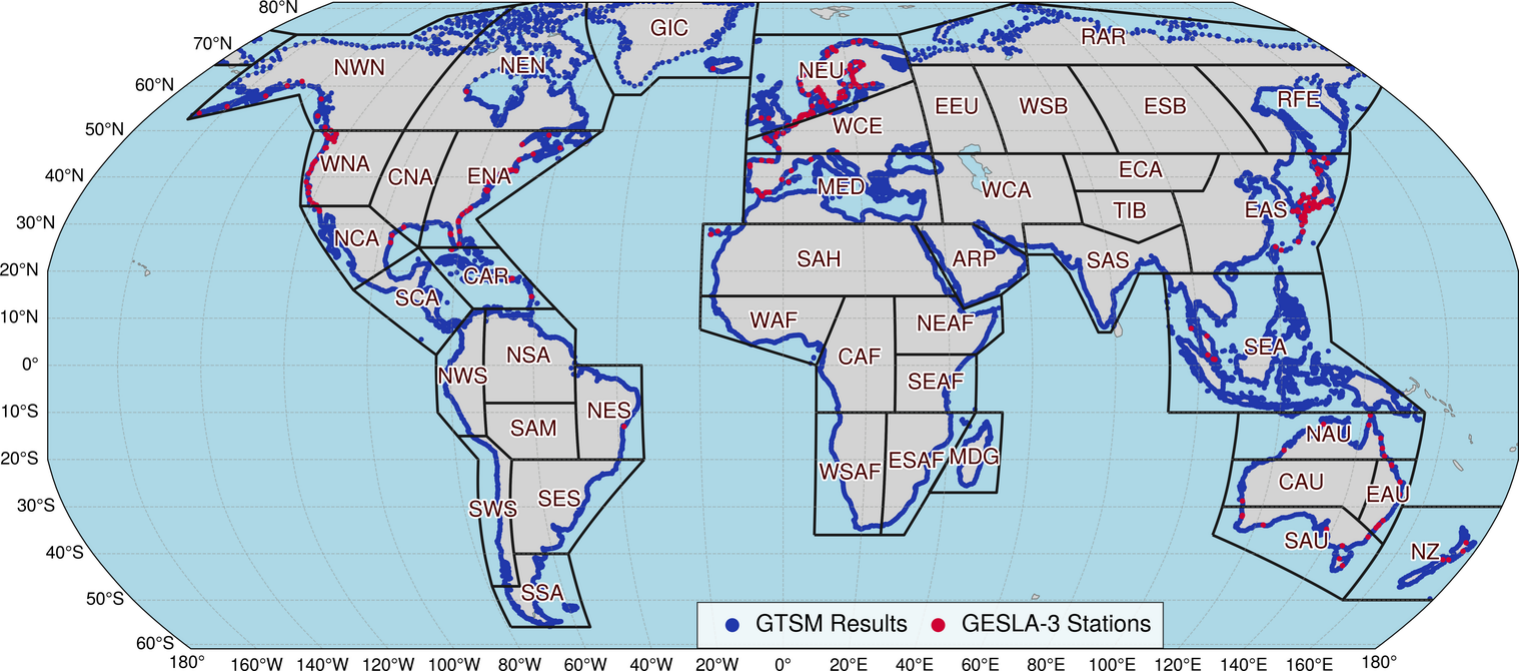
Global distribution of GTSM output points (blue) and GESLA-3 stations (red) along coastlines within IPCC reference regions

**Fig. 3:**
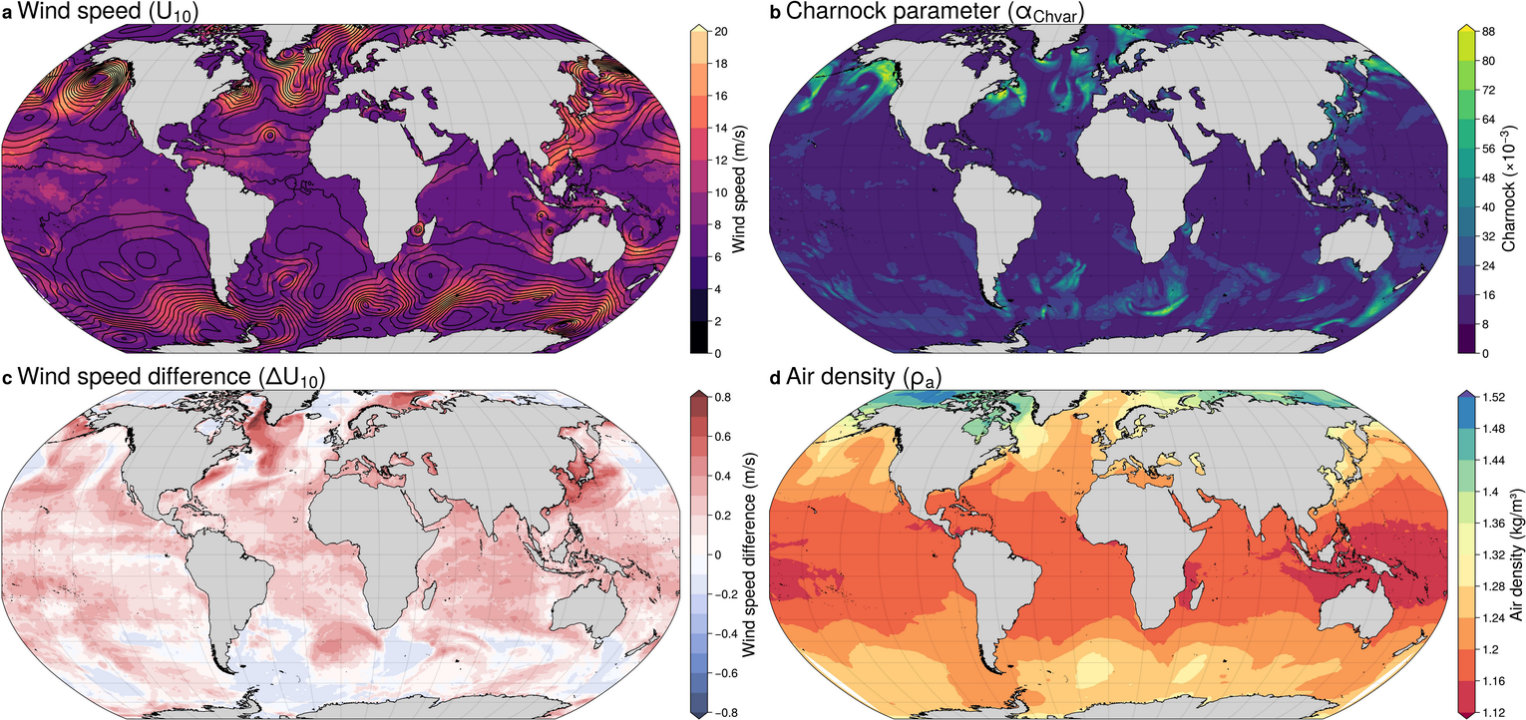
Global distributions of parameters on January 1, 2008 at 00 UTC, using ERA5 data: **a** Wind speed at 10 m ($U_{10}$, colours) and mean sea level pressure (contours at 4 hPa intervals); **b** Charnock parameter ($\alpha _{\text {Chvar}} \times 10^{-3}$); **c** Wind speed difference ($\Delta U_{10} = U_{10N} \mathbf {-} U_{10}$); **d** Air density ($\rho _a$) calculated using ERA5 parameters

### Observational data

For validation of the wind stress parameterization, we compare our different model experiments with observational data from tide gauge records, using the Global Extreme Sea Level Analysis Version 3.0 (GESLA-3) dataset (Haigh et al. 2022) and data from Fisheries and Oceans Canada (DFO, 2023). Before this comparison, we apply a series of data processing and filtering steps for GESLA-3. These steps include: (i) selecting only ‘coastal’ tide gauge stations and prioritizing those marked as having ‘no obvious issues’ based on record quality; (ii) managing potential duplicates by retaining only the longest data record when multiple records exist for the same site and when stations are within three kilometers of each other; (iii) standardizing the dataset to hourly intervals (instantaneous) to ensure consistency in the analysis; (iv) ensuring that the resampled data maintain a minimum of 90% hourly data availability per year for each station. Additionally, for each station water levels are adjusted by subtracting the overall mean and annual means to remove long-term variations, aligning with the outputs of GTSM mean sea level. However, factors such as ocean temperature variations, salinity changes, and currents, which often lead to low-frequency fluctuations in mean sea level, are not incorporated in GTSM. While these low-frequency fluctuations are present in the observational data but not modeled in GTSM, no specific alignment method has been applied to address these discrepancies.

Following the data processing and filtering steps, we obtain a dataset consisting of observations from 300 stations, with data spanning from 2006 to 2015 at hourly intervals from GESLA-3. Next, using this data in addition to the dataset from Fisheries and Oceans Canada (DFO, 2023), we derive the surge residuals by subtracting the harmonic tide from the observed total water for each tide gauge station. Consistent with our treatment of the model results, the harmonic tide was calculated using the hatyan software (Veenstra 2022).

Figure 2 illustrates GTSM output points and GESLA-3 observational stations, including the Intergovernmental Panel on Climate Change (IPCC) reference regions (Iturbide et al. 2020). These GESLA-3 stations are distributed across many coastal zones, but regions such as Africa and the southern Pacific lack coverage. Of the 41 coastal IPCC regions (excluding landlocked areas), approximately 21 regions have at least one tide gauge station, while only 6 regions have more than ten stations.

## Results

### Global variability of parameters

This section examines the spatial variability of key atmospheric parameters influencing air-sea interactions, including the actual wind speed at 10 m ($U_{10}$), the Charnock parameter ($\alpha _{\text {Chvar}}$), the air density ($\rho _a$), and the difference between the neutral wind speed and the actual wind speed at 10 m, defined as $\Delta U_{10} = U_{10N} \mathbf {-} U_{10}$. The time snapshot shown in Fig. 3 is randomly chosen to illustrate the spatial distribution of these parameters within a single time step.

The actual wind speeds at 10 m above the sea surface show considerable spatial variability, with the highest values exceeding 20 m/s concentrated in mid-latitude regions such as the Southern Ocean, North Atlantic, and North Pacific. These regions, where atmospheric pressure gradients drive intense winds, are critical for transferring energy from the atmosphere to the ocean, contributing to wave generation, surface mixing, and storm surges. The near-equatorial region shows lower wind speeds, generally below 6 m/s, highlighting the calmer atmospheric conditions over the tropical regions (Fig. 3a).

To better understand how sea-state-dependent roughness varies spatially, we examine the global distribution of the Charnock parameter from IFS. The Charnock parameter, as computed by the wave model in IFS, varies significantly across the globe, ranging from a minimum of $8 \times 10^{-3}$ to over $80 \times 10^{-3}$. The highest values are observed in regions with strong winds, such as the Southern Ocean, where they frequently exceed $60 \times 10^{-3}$, and the North Atlantic during extra-tropical cyclone activity, with values between $40$–$80 \times 10^{-3}$ (Fig. 3b). These elevated values are driven by intense winds and young wave conditions, which increase surface roughness and amplify wind stress, contributing to storm surges. In contrast, tropical regions like the central Pacific and Indian Ocean show much lower Charnock values, generally below $20 \times 10^{-3}$, reflecting weaker winds and calmer sea states. Similarly, in areas dominated by older, long-period waves, such as the southern Indian Ocean, values remain relatively low (typically $10$–$30 \times 10^{-3}$), even under moderate to strong winds. By comparison, extra-tropical cyclones in mid-latitudes, such as those in the North Atlantic, generate steep, young waves that elevate Charnock values above $50 \times 10^{-3}$. When the atmospheric model in IFS is run without coupling to the ocean model, the Charnock parameter is fixed at $0.018$ (or $18 \times 10^{-3}$) (ECMWF 2023a), which fits moderate wind speeds but underestimates surface roughness during storms or in high-wind regions. Additionally, the default Charnock value in GTSM is set to $0.041$ (or $41\times 10^{-3}$), which better represents average surface roughness for stronger winds. While this value aligns more closely with the observed Charnock parameter in regions with moderate-to-high winds, such as the mid-latitudes, it falls short of capturing the full variability during extreme conditions, particularly in areas like the Southern Ocean or the North Atlantic during extra-tropical cyclones.

Figure 3c depicts the global distribution of the wind speed difference, with values ranging from -0.8 m/s to +0.8 m/s. Neutral wind speeds are on average about 0.5 m/s stronger than actual wind speeds across most of the global oceans, highlighting the widespread presence of unstable marine boundary layers (Archer et al. 2016). Positive differences, where neutral wind speed exceeds actual wind speed, are prevalent in tropical and subtropical regions. These areas, characterized by warm sea surface temperatures, experience strong vertical mixing that reduces surface wind gradients, resulting in differences typically ranging from +0.1 m/s to +0.8 m/s. Approximately 30–40% of the ocean areas show differences greater than +0.5 m/s. Since wind stress scales approximately with the square of wind speed, a difference of 0.8 m/s at a background wind speed of 8 m/s can lead to a change in stress of about 20%. In contrast, negative differences, where actual wind speeds exceed neutral wind speeds, are more localized and tend to occur in higher latitudes and coastal regions. Here, warmer air above the ocean surface reduces vertical mixing, causing differences between -0.1 m/s to -0.8 m/s. About 10–20% of the oceans show differences below -0.5 m/s. Regions with minimal differences ($|\Delta U_{10}| \le 0.05 \, \mathrm {m/s}$) are typically found in areas with weak temperature gradients, such as transitional zones. Finally, in high-wind regions like the North Atlantic and Southern Ocean, extra-tropical cyclones enhance vertical mixing and bring the boundary layer closer to neutral stability, resulting in smaller differences, often below 0.3 m/s.

Finally, air density also shows significant spatial variability, ranging from approximately 1.12 to 1.48 kg/m$^3$ (Fig. 3d). Polar regions, such as the Southern Ocean and the Arctic, show higher air densities (1.36–1.48 kg/m$^3$) due to colder temperatures, while tropical regions near the equator have lower densities, often below 1.20 kg/m$^3$. This latitude-dependent variation reflects the temperature-driven relationship with air density, where colder air is denser and warmer air is less dense. The largest deviations from the constant default value of 1.20 kg/m$^3$ occur in the Arctic, where densities are nearly 25% higher, reaching up to 1.48 kg/m$^3$, while the lowest values in tropical regions are about 10% lower (around 1.12 kg/m$^3$). It is also important to note that the snapshot date (January 1) corresponds to summer in the Southern Hemisphere and winter in the Northern Hemisphere, which explains why air density is relatively higher in the Arctic and lower in the Antarctic. Since air density appears as a linear factor in the wind stress formulation, such variations can have a direct and substantial impact on storm surge forcing. Lower air densities in the tropics may reduce wind stress, resulting in the underestimation of storm surges compared to the model’s default, while higher densities in polar regions amplify wind stress for the same wind speed, increasing storm surge potential.

**Fig. 4:**
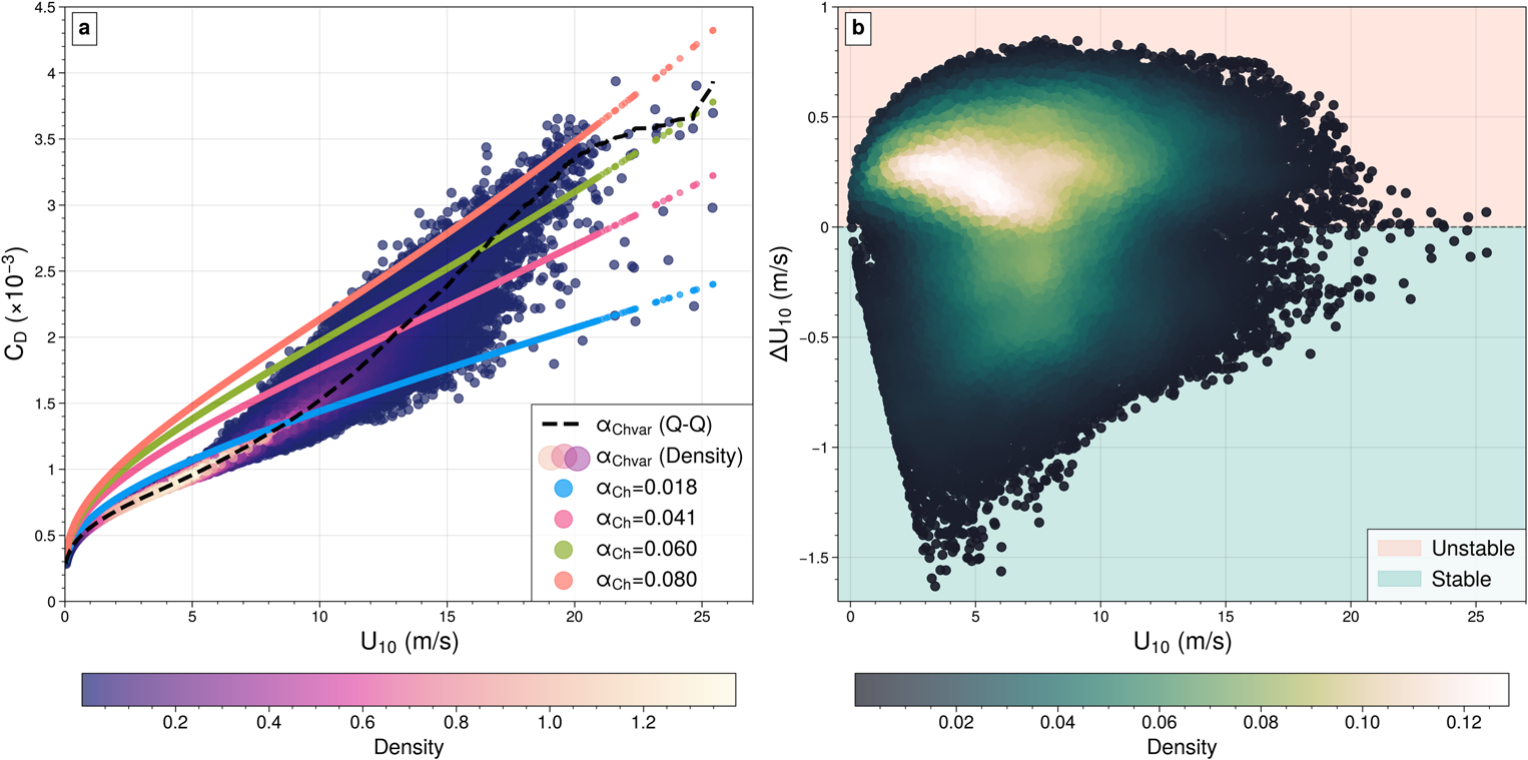
**a** Drag coefficient ($C_D \times 10^{-3}$) as a function of 10-m wind speed ($U_{10}$, calculated using both constant and variable Charnock parameters ($\alpha _{\text {Ch}}$). Solid lines represent fixed Charnock ($\alpha _{\text {Ch}}$): blue ($0.018$), pink ($0.041$), green ($0.060$), and orange ($0.080$). The variable Charnock parameter ($\alpha _{\text {Chvar}}$) is illustrated by the density plot (shaded area) and Q-Q (quantile-quantile) method (dashed black line). **b** Wind speed difference ($\Delta U_{10}$) as a function of 10-m wind speed ($U_{10}$), with scatter points shaded by density. The background highlights regions of unstable (peach) and stable (light green) boundary layer conditions. Both **a** and **b** data span the period from 2006 to 2015 at a point in the North Atlantic

Figure 4 illustrates the influence of sea surface roughness on the drag coefficient ($C_D$) across different Charnock parameters (Fig. 4a), and the relationship between $U_{10}$ and $\Delta U_{10}$ (Fig. 4b) at a location in the North Atlantic. Fixed Charnock values are represented by the colored lines, where higher values (e.g., $\alpha _{\text {Ch}} = 0.080$) correspond to rougher ocean surfaces and result in higher drag coefficients for the same wind speed, while lower values (e.g., $\alpha _{\text {Ch}} = 0.018$) represent smoother surfaces with less drag (Fig. 4a). The black dashed line represents a variable Charnock parameterization using the Q-Q (quantile-quantile) approach. At moderate wind speeds ($<10$ m/s), it aligns closely with a fixed Charnock value of 0.018, indicative of calmer seas. As wind speeds rise ($>15$ m/s), it approaches a value of 0.060, reflecting the transition to rougher sea states (Fig. 4a). This adaptability makes the variable parameterization well-suited for dynamic conditions, such as extreme weather events, where sea surface roughness evolves rapidly with changing wind speeds. The scatter points, shaded by density, show that most data cluster at moderate wind speeds, while greater variability emerges at higher wind speeds, which becomes particularly important for storm surge modelling (Fig. 4a).

Of particular interest is the pink line ($\alpha _{\text {Ch}} = 0.041$), previously used as the default Charnock parameter in our GTSM model (Dullaart et al. 2020; Muis et al. 2016, 2019, 2020). This value, derived from regional studies in the North Sea (F. Zijl, personal communication, November, 2022), represents ocean roughness under moderately turbulent conditions and balances momentum transfer across a wide range of wind speeds. While $\alpha _{\text {Ch}} = 0.018$ may adequately describe surface roughness at moderate wind speeds, it is likely to underestimate drag in high wind conditions, such as storms (Fig. 4a). In contrast, the default value of $\alpha _{\text {Ch}} = 0.041$ provides a more appropriate baseline, ensuring that wind-generated waves and surface stress are better captured at higher wind speeds (Fig. 4a).

Furthermore, Fig. 4b illustrates the relationship between $U_{10}$ and $\Delta U_{10}$, highlighting the influence of atmospheric stability. On average, the atmospheric boundary layer is slightly unstable ($\Delta U_{10} > 0$), with the highest density of data points occurring around $U_{10} \approx 5-10~\mathrm {m/s}$ and $\Delta U_{10} \approx 0.2~m/s$, indicating near-neutral to slightly unstable conditions. This pattern is also observed in Hersbach (2008); Kara et al. (2008), which report similar results. Stable conditions ($\Delta U_{10} < 0$) are also frequent at lower wind speeds ($U_{10}<5$ m/s), with weaker turbulence and reduced mixing. As wind speeds increase beyond 15 m/s, the effects of stability diminish due to enhanced mechanical mixing, resulting in a narrower spread of $\Delta U_{10}$.

### Evaluation of storm events

This section evaluates two individual storm events to assess the model’s performance under extreme conditions. The analysis begins with the Xaver storm event (December 2013), followed by the Fiona storm event (September 2022).

**Fig. 5:**
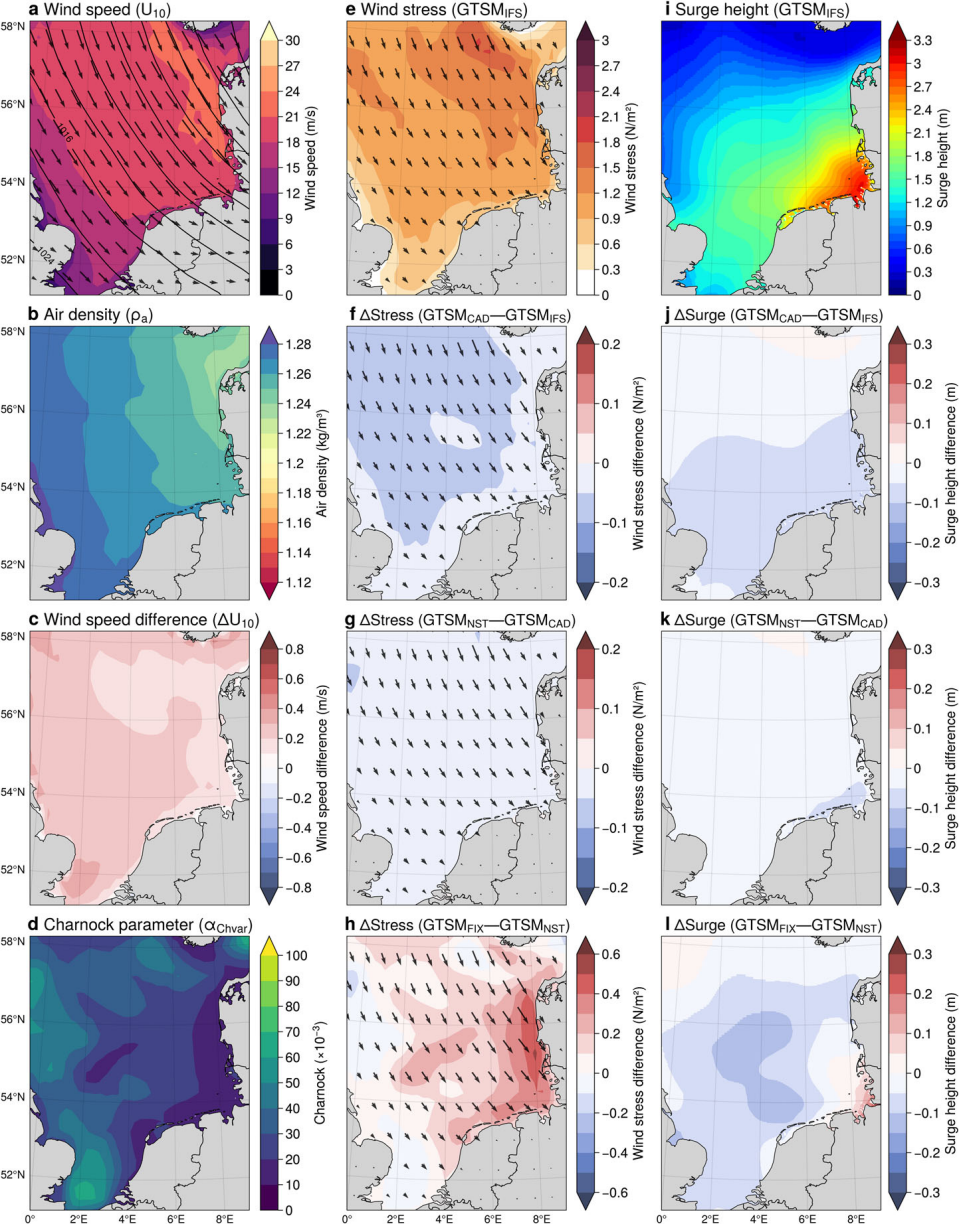
Storm Xaver analysis on December 6, 2013, at 05 UTC, using ERA5: **a** Wind speed ($U_{10}$, colours/arrows) and mean sea level pressure (contours); **b** Air density ($\rho _a$); **c** Wind speed difference ($\Delta U_{10}$); **d** Charnock parameter ($\alpha _{\text {Chvar}}\times 10^{-3}$); **e** Wind stress ($\text {GTSM}_{\text {IFS}}$); **f–h** Wind stress differences ($\Delta Stress$) and directions: $\text {GTSM}_{\text {CAD}}-\text {GTSM}_{\text {IFS}}$, $\text {GTSM}_{\text {NST}}-\text {GTSM}_{\text {CAD}}$, $\text {GTSM}_{\text {FIX}}-\text {GTSM}_{\text {NST}}$; **i** Surge height ($\text {GTSM}_{\text {IFS}}$); **j–l** Surge height differences ($\Delta Surge$): $\text {GTSM}_{\text {CAD}}-\text {GTSM}_{\text {IFS}}$, $\text {GTSM}_{\text {NST}}-\text {GTSM}_{\text {CAD}}$, $\text {GTSM}_{\text {FIX}}-\text {GTSM}_{\text {NST}}$

#### Storm xaver

Figure 5 presents an analysis of Storm Xaver on December 6, 2013, at 05 UTC, using ERA5 reanalysis data and GTSM experiments. Wind speeds at 10 meters ($U_{10}$), shown in Fig. 5a, exceed 24 m/s in the northern North Sea, with strong northwesterly winds dominating the region. Corresponding air density ($\rho _a$) varies between 1.22 kg/m$^3$ and 1.28 kg/m$^3$ (Fig. 5b), reflecting cold atmospheric conditions typical of winter storms in the North Sea. Most severe storms in the North Sea occur during the winter months, when air temperatures lower and air density higher. Neutral winds are up to 0.4 m/s stronger than actual winds in some area, suggesting the presence of an unstable atmosphere (Fig. 5c). The Charnock parameter ($\alpha _{\text {Chvar}}$), which controls surface roughness, varies from 10 to 70 $\times 10^{-3}$, with higher values in areas of active wave growth and lower values in older sea states near the coast (Fig. 5d). Additionally, Fig. 5e illustrates the wind stress field produced by the $\text {GTSM}_{\text {IFS}}$ experiment, which includes the effects of spatially varying air density, atmospheric stability, and sea-state-dependent drag. Wind stress magnitudes exceed 2.1 N/m$^2$ in regions where wind speeds and surface roughness are highest, particularly over the northern North Sea. In contrast, wind stress values near the northern coastline of Germany are lower, around 1.2 N/m$^2$, due to an older sea state that reduces wave-dependent drag. The resulting surge heights from the $\text {GTSM}_{\text {IFS}}$ experiment (Fig. 5i) exceed 3.2 m near the northern German coast. The strong wind-driven surge aligns with the transport of colder air masses from higher latitudes, which typically creates an unstable atmosphere and enhances wind stress over the central North Sea (Mo et al. 2016). However, the strongest winds remain offshore at the time of this snapshot, limiting the immediate coastal impact. Although Fig. 5 captures the peak surge (December 6 at 05 UTC), the observed surge levels are the result of wind stress accumulated over the preceding hours. For instance, wind stress was stronger earlier in the storm (e.g., December 5 at 18 UTC, not shown), contributing to coastal surge buildup.

**Fig. 6:**
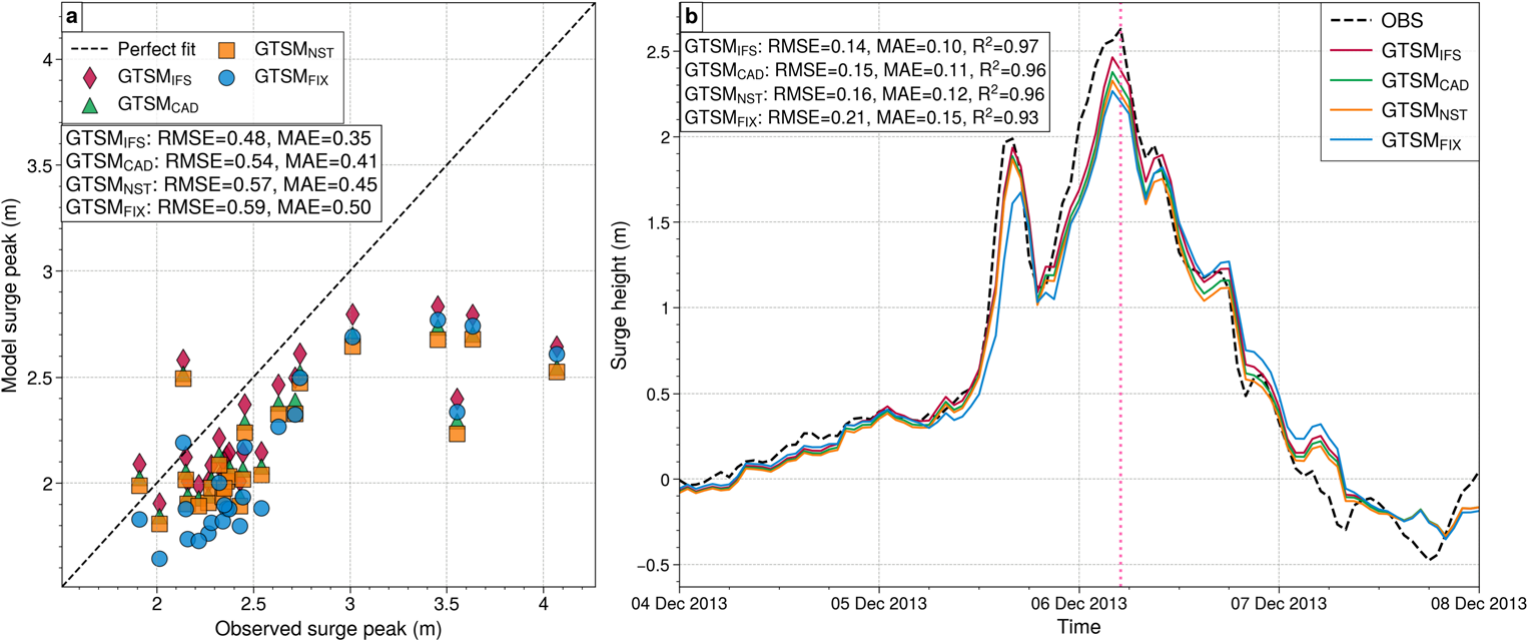
**a** Scatter plot comparing observed and modeled surge peaks from 25 tide gauge stations for GTSM experiments: $\text {GTSM}_{\text {IFS}}$ (red diamonds), $\text {GTSM}_{\text {CAD}}$ (green triangles), $\text {GTSM}_{\text {NST}}$ (orange squares), and $\text {GTSM}_{\text {FIX}}$ (blue circles). The dashed black line represents a perfect fit. Root mean square error (RMSE) and mean absolute error (MAE) are shown for each experiment. **b** Time series of surge heights at Huibertgat showing observed data (OBS, black dashed line) alongside results from GTSM experiments: $\text {GTSM}_{\text {IFS}}$ (red), $\text {GTSM}_{\text {CAD}}$ (green), $\text {GTSM}_{\text {NST}}$ (orange), and $\text {GTSM}_{\text {FIX}}$ (blue). RMSE, MAE and $R^{2}$ values are included for each experiment

To evaluate the effects of different sea surface drag, we compare wind stress and storm surge between the GTSM experiments. Wind stress and storm surge differences between GTSM$_{\text {CAD}}$-GTSM$_{\text {IFS}}$ and GTSM$_{\text {NST}}$-GTSM$_{\text {CAD}}$ are relatively minor, indicating that air density variations and atmospheric stability have only a limited effect on the modeled storm surges. Figure 5f shows that wind stress in GTSM$_{\text {CAD}}$ is approximately 0.1 N/m$^2$ lower than in GTSM$_{\text {IFS}}$, confirming that assuming a constant air density slightly underestimates wind stress. However, since the largest air density variations occur over colder offshore regions where the wind stress is already high, their influence on overall surge generation is minimal. Similarly, Fig. 5g shows that switching from neutral to actual winds in GTSM$_{\text {NST}}$ results in a further slight reduction in wind stress. These small reductions in wind stress translate into minor surge differences, as seen in Fig. 5j and k, where surge heights are generally 0.05 to 0.1 m lower in GTSM$_{\text {CAD}}$ compared to GTSM$_{\text {IFS}}$ and in GTSM$_{\text {NST}}$ compared to GTSM$_{\text {CAD}}$. These results indicate that while air density variations and atmospheric stability are accounted for, their overall impact on wind stress and surge height remains relatively small. In contrast, changing from a sea-state-dependent to a constant Charnock parameter has a more pronounced effect on wind stress and storm surge. Figure 5h shows that GTSM$_{\text {FIX}}$ overestimates wind stress by up to 0.6 N/m$^2$ compared to GTSM$_{\text {NST}}$, which accounts for wave-state-dependent roughness. This difference is most evident in the central North Sea, where the older sea state leads to reduced wave-dependent drag (Fig. 5d), resulting in lower wind stress in GTSM$_{\text {NST}}$. Consequently, Fig. 5l shows surge differences with a slight increase (around 0.20 m) in the central North Sea under GTSM$_{\text {NST}}$, while a notable decrease of 0.20 m occurs along the northern German coastline due to lower wind stress in this region (Fig. 5l).

**Fig. 7:**
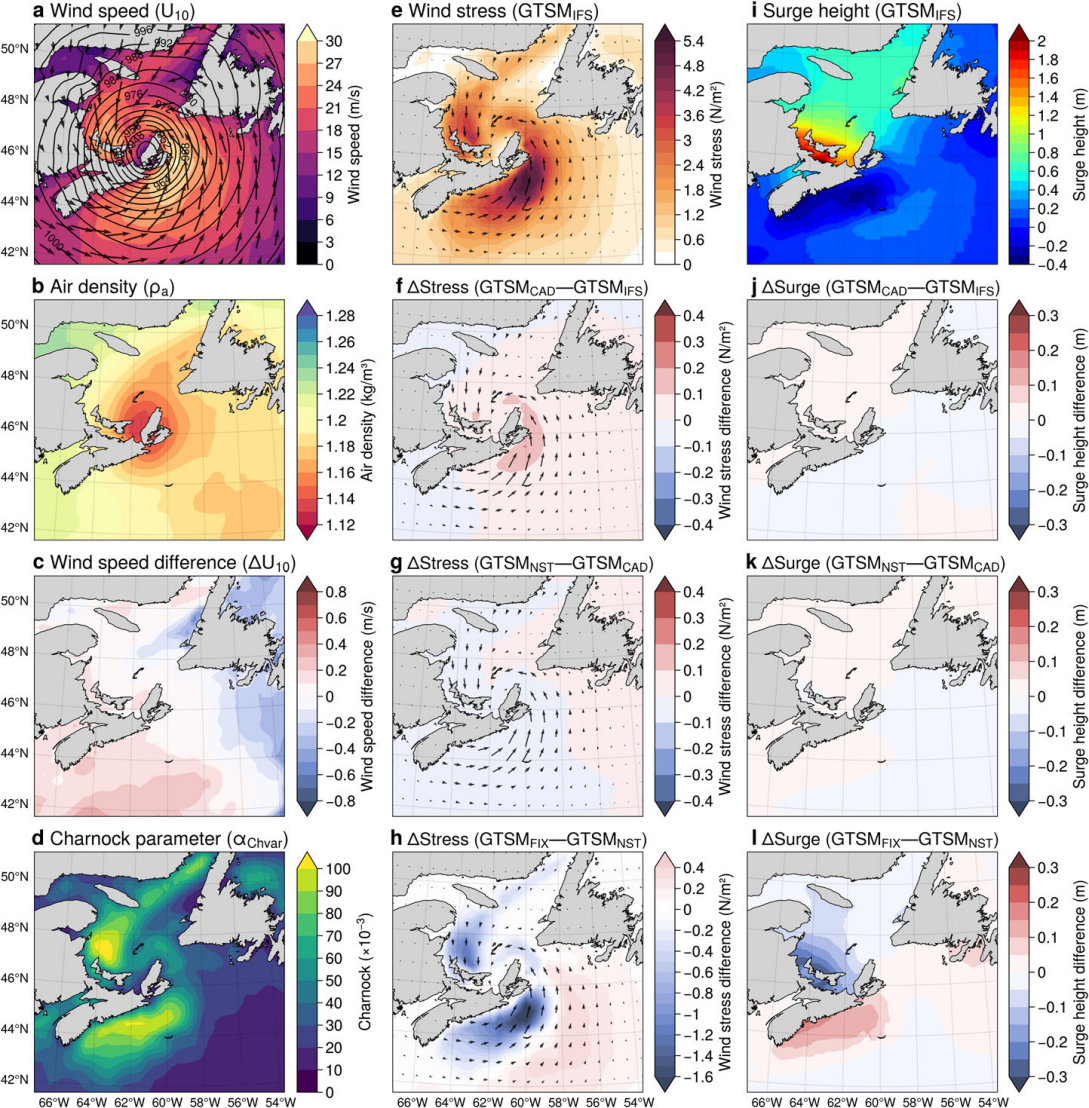
Hurricane Fiona analysis on September 24, 2022 at 10 UTC, using ERA5: **a** Wind speed ($U_{10}$, colours/arrows) and mean sea level pressure (contours); **b** Air density ($\rho _a$); **c** Wind speed difference ($\Delta U_{10}$); **d** Charnock parameter ($\alpha _{\text {Chvar}}\times 10^{-3}$); **e** Wind stress ($\text {GTSM}_{\text {IFS}}$); **f–h** Wind stress differences ($\Delta Stress$) and directions: $\text {GTSM}_{\text {CAD}}-\text {GTSM}_{\text {IFS}}$, $\text {GTSM}_{\text {NST}}-\text {GTSM}_{\text {CAD}}$, $\text {GTSM}_{\text {FIX}}-\text {GTSM}_{\text {NST}}$; **i** Surge height ($\text {GTSM}_{\text {IFS}}$); **j–l** Surge height differences ($\Delta Surge$): $\text {GTSM}_{\text {CAD}}-\text {GTSM}_{\text {IFS}}$, $\text {GTSM}_{\text {NST}}-\text {GTSM}_{\text {CAD}}$, $\text {GTSM}_{\text {FIX}}-\text {GTSM}_{\text {NST}}$

Figure 6 compares observed and modeled surge heights during Storm Xaver for four GTSM experiments. Figure 6a shows maximum surge heights at 25 tide gauges along the Dutch coast, while Fig. 6b presents the surge height time series at Huibertgat station. The $\text {GTSM}_{\text {IFS}}$ experiment demonstrates the best agreement with observations, achieving the lowest RMSE (0.48 m) and MAE (0.35 m), while $\text {GTSM}_{\text {CAD}}$ and $\text {GTSM}_{\text {NST}}$ follow closely with RMSE values of 0.54 m and 0.57 m, respectively (Fig. 6a). The $\text {GTSM}_{\text {FIX}}$ shows the largest errors (RMSE = 0.59 m, MAE = 0.50 m) and usually underestimates. Additionally, all experiments show strong correlations with observations, with $R^2$ values ranging from 0.93 to 0.97 at Huibertgat (Fig. 6b). Among the experiments, $\text {GTSM}_{\text {IFS}}$ performs best, achieving an RMSE of 0.14 m, followed by $\text {GTSM}_{\text {CAD}}$ (0.15 m) and $\text {GTSM}_{\text {NST}}$ (0.16 m), while the $\text {GTSM}_{\text {FIX}}$ shows the highest error (0.21 m). All four experiments successfully capture the two prominent surge events on 5th December at 16 UTC and 6th December at 4 UTC. For the first event, $\text {GTSM}_{\text {IFS}}$ provides the closest match (approximately 1.9 m), followed by $\text {GTSM}_{\text {CAD}}$ and $\text {GTSM}_{\text {NST}}$ (both approximately 1.85 m), and $\text {GTSM}_{\text {FIX}}$ (approximately 1.65 m). Similarly, for the second event, $\text {GTSM}_{\text {IFS}}$ predicts approximately 2.48 m, for the observed surge peak of 2.62 m, while the other experiments slightly underestimate it: approximately 2.35 m ($\text {GTSM}_{\text {CAD}}$), 2.30 m ($\text {GTSM}_{\text {NST}}$), and 2.25 m ($\text {GTSM}_{\text {FIX}}$). These results demonstrate that $\text {GTSM}_{\text {IFS}}$ slightly gives more accurate predictions, highlighting the benefits of incorporating variable air density, stability and surface roughness in improving GTSM’s performance. Each addition to the stress parameterization, including the sea-state-dependent Charnock, atmospheric stability correction, and variable air density, contributes to this improvement.

**Fig. 8:**
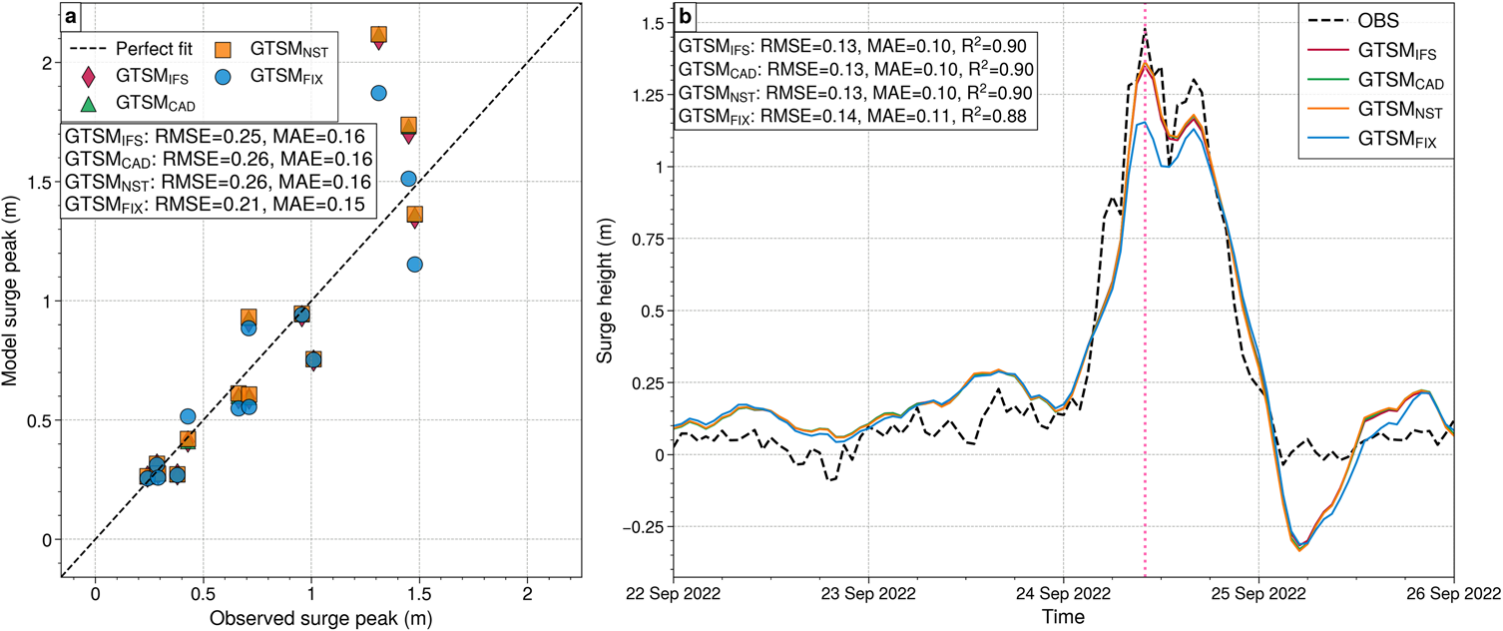
**a** Scatter plot comparing observed and modeled surge peaks from 13 tide gauge stations for GTSM experiments: $\text {GTSM}_{\text {IFS}}$ (red diamonds), $\text {GTSM}_{\text {CAD}}$ (green triangles), $\text {GTSM}_{\text {NST}}$ (orange squares), and $\text {GTSM}_{\text {FIX}}$ (blue circles). The dashed black line represents a perfect fit. Root mean square error (RMSE) and mean absolute error (MAE) are shown for each experiment. **b** Time series of surge heights at Lower Escuminac showing observed data (OBS, black dashed line) alongside results from GTSM experiments: $\text {GTSM}_{\text {IFS}}$ (red), $\text {GTSM}_{\text {CAD}}$ (green), $\text {GTSM}_{\text {NST}}$ (orange), and $\text {GTSM}_{\text {FIX}}$ (blue). RMSE, MAE and $R^{2}$ values are included for each experiment

#### Hurricane Fiona

Figure 7 examines Hurricane Fiona at 03 UTC on September 24, 2022, using ERA5 reanalysis and outputs from four GTSM experiments. At this stage, Hurricane Fiona had transitioned into a post-tropical cyclone centered over Nova Scotia, with a powerful wind field extending far from the storm’s core. The storm exhibited a well-defined cyclonic circulation over the Gulf of St. Lawrence, with wind speeds exceeding 30 m/s, particularly near the coastlines of Newfoundland and Nova Scotia (Fig. 7a). The air density field shows lower values of 1.12–1.16 kg/m$^3$ near the storm center, where the lowest atmospheric pressure is found, while higher densities of 1.20–1.24 kg/m$^3$ appear farther from the core (Fig. 7b). The low air density near the center is characteristic of extreme low-pressure systems, which are associated with warmer, less dense air. The difference between neutral wind speed and actual wind speed indicates that stronger winds lead to a more neutrally stratified atmosphere (Fig. 7c). Surface roughness, represented by the Charnock parameter in Fig. 7d, is highest in regions with strong winds, exceeding $100 \times 10^{-3}$ near the coastlines of Nova Scotia. This indicates a young sea state, where strong winds generate developing waves, increasing surface drag and enhancing wind stress. In addition, the wind stress field in Fig. 7e is derived from the $\text {GTSM}_{\text {IFS}}$ experiment, which resolves spatial variations in air density, atmospheric stability and wave-dependent drag. The highest wind stress values exceed 5.4 N/m$^2$, particularly where the strongest winds and roughest sea states are located. This stress distribution closely follows the storm’s cyclonic wind field, with strong gradients near the coasts of Newfoundland and Nova Scotia. The corresponding storm surge in $\text {GTSM}_{\text {IFS}}$ exceeds 2.0 m in the southern Gulf of St. Lawrence, where onshore winds push water toward the coast (Fig. 7i). On the other hand, along the southern coast of Nova Scotia, offshore winds result in a negative surge of about -0.2 m, as water is displaced away from the shore.

We evaluate the impact of varying sea surface drag representations by comparing wind stress and storm surge across the GTSM experiments. Figure 7f highlights the effect of using a constant air density, showing the wind stress difference between $\text {GTSM}_{\text {CAD}}$ and $\text {GTSM}_{\text {IFS}}$. Since wind stress is directly proportional to air density, the assumption of a constant value of 1.20 kg/m$^3$ in $\text {GTSM}_{\text {CAD}}$ results in higher wind stress (by up to 0.2 N/m$^2$) near the storm center, where $\text {GTSM}_{\text {IFS}}$ accounts for lower air density. The impact on storm surge remains minimal, as reflected in Fig. 7j, where surge differences remain below 0.1 m. This suggests that while accounting for air density variations improves physical consistency, it has little effect on storm surge predictions. Similarly, Fig. 7g shows that the impact of atmospheric stability ($\text {GTSM}_{\text {NST}}$–$\text {GTSM}_{\text {CAD}}$) on wind stress is minimal (differences below 0.1 N/m$^2$), with near-zero surge differences in Fig. 7k, confirming its limited influence on storm surge response. Conversely, applying a fixed Charnock parameter instead of a dynamic, sea-state-based one leads to stronger changes in both wind stress and storm surge. As shown in Fig. 7h, $\text {GTSM}_{\text {FIX}}$, which uses a fixed roughness length, underestimates wind stress by up to 1.6 N/m$^2$ compared to $\text {GTSM}_{\text {NST}}$, particularly along the south and north coasts of Nova Scotia where wave-state-dependent roughness enhances wind stress. As shown in Fig. 7d, a young sea state enhances surface drag, leading to higher Charnock values and consequently stronger wind stress. The differences between $\text {GTSM}_{\text {FIX}}$ and $\text {GTSM}_{\text {NST}}$ (Fig. 7h) indicate that using a constant Charnock parameter leads to lower wind stresses than dynamically varying roughness, which in turn results in a decrease in surge height, down to 0.30 m along Northern Nova Scotia, southeastern New Brunswick, and northern Prince Edward Island (Fig. 7l) but higher surges up to 0.30 m along southern coast of Nova Scotia. This lowers the total storm surge height to approximately 1.8 m along Northern Nova Scotia coast.

**Fig. 9:**
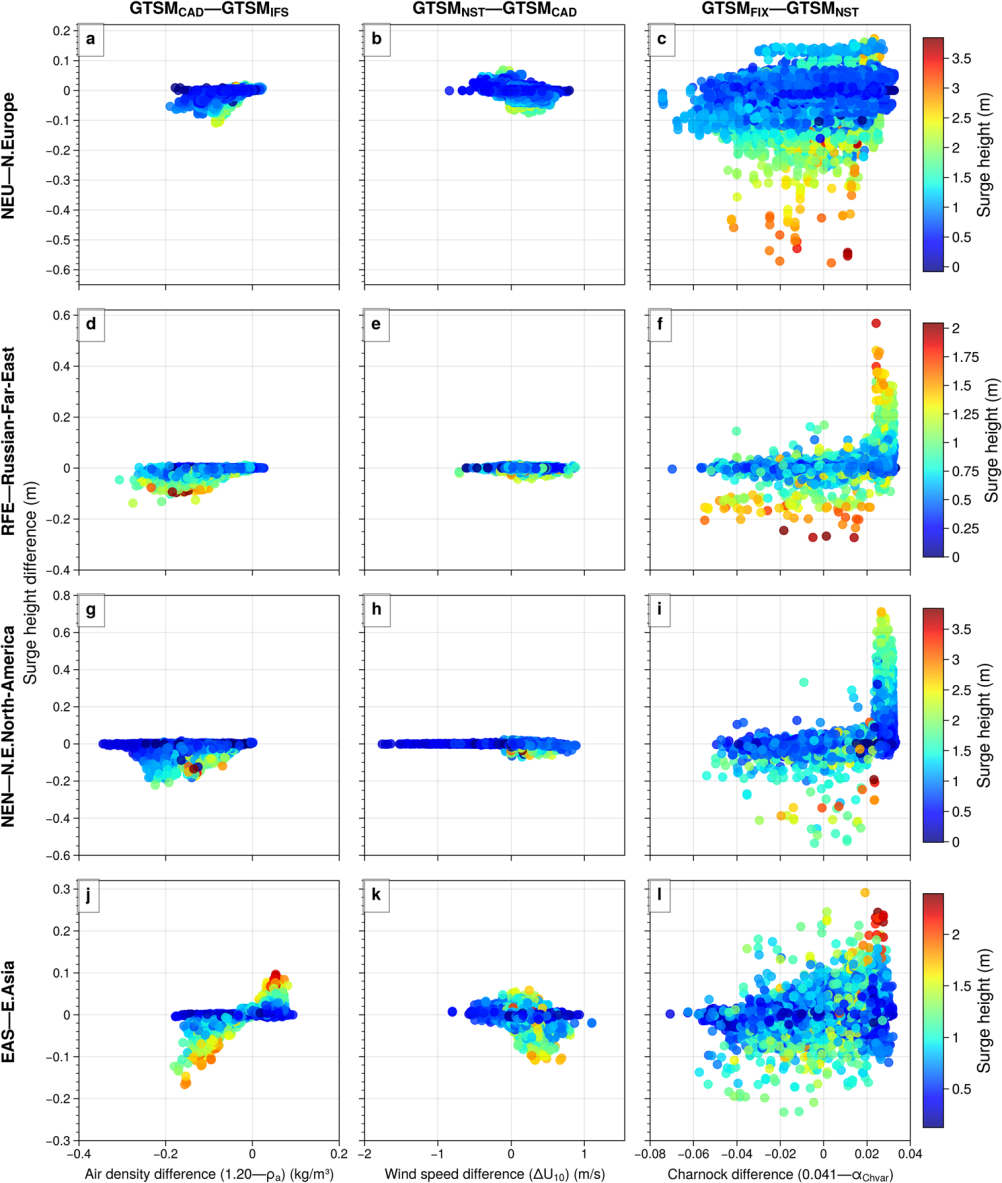
Regional comparison of annual maximum surge height differences for 2006–2015, based on changes in air density (1.20 - $\rho _a$), wind speed ($\Delta U_{10}$) and Charnock parameter (0.041 - $\alpha _{\text {Chvar}}$). Each column represents differences between model experiments: **a–j** $\text {GTSM}_{\text {CAD}} - \text {GTSM}_{\text {IFS}}$; **b–k** $\text {GTSM}_{\text {NST}} - \text {GTSM}_{\text {CAD}}$; **c–l** $\text {GTSM}_{\text {FIX}} - \text {GTSM}_{\text {NST}}$. Rows correspond to different regions: **a–c** NEU (Northern Europe, $n = 36460$); **d–f** RFE (Russian Far East, $n = 3570$); **g–i** NEN (Northeast North America, $n = 5290$); **j–l** EAS (East Asia, $n = 8960$). Point colors indicate surge height magnitudes

**Fig. 10:**
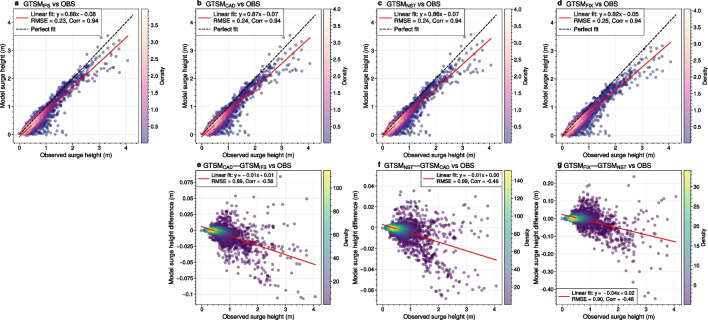
Global comparison of annual maximum surge heights between model predictions and observations (OBS) across 300 stations for 2006-2015. **a–d**: Model predictions ($\text {GTSM}_{\text {IFS}}, \text {GTSM}_{\text {CAD}}, \text {GTSM}_{\text {NST}}, \text {GTSM}_{\text {FIX}}$) vs OBS; **e–g**: Residual differences between models ($\text {GTSM}_{\text {CAD}}-\text {GTSM}_{\text {IFS}}$, $\text {GTSM}_{\text {NST}}-\text {GTSM}_{\text {CAD}}$, $\text {GTSM}_{\text {FIX}}-\text {GTSM}_{\text {NST}}$) vs OBS

Figure 8 compares observed and modeled surge heights during Hurricane Fiona for four GTSM experiments. Figure 8a compares maximum observed and modeled surge peaks at 13 tide gauges along Canada’s eastern coast. Figure 8b shows the surge height time series at Lower Escuminac station during the hurricane. $\text {GTSM}_{\text {FIX}}$ shows the best agreement with observations, achieving the lowest RMSE (0.21 m) and MAE (0.15 m), while $\text {GTSM}_{\text {IFS}}$, $\text {GTSM}_{\text {NST}}$, and $\text {GTSM}_{\text {CAD}}$ follow with similar performance, each recording RMSE values of 0.25–0.26 m and MAE values of 0.16 m (Fig. 8a). Additionally, all experiments indicate strong correlations with observations, with $R^2$ values consistently around 0.90 (Fig. 8b). Among the experiments, $\text {GTSM}_{\text {IFS}}$, $\text {GTSM}_{\text {NST}}$, and $\text {GTSM}_{\text {CAD}}$ demonstrate slightly better performance, each achieving an RMSE of 0.13 m and an MAE of 0.10 m, while $\text {GTSM}_{\text {FIX}}$ follows closely with an RMSE of 0.14 m and an MAE of 0.11 m. All four experiments effectively capture the prominent surge event on 24th September 2022 at 10 UTC, when the observed surge peaked at approximately 1.5 m. For this event, $\text {GTSM}_{\text {IFS}}$, $\text {GTSM}_{\text {CAD}}$ and $\text {GTSM}_{\text {NST}}$ provide the closest match to the observed data surge peaked value at approximately 1.3 m, while $\text {GTSM}_{\text {FIX}}$ underestimates more with surge peak value of 1.1 m.

### Evaluation of model performance and regional surge height variability

Figure 9 provides a regional comparison of surge height differences across four model experiments, using annual maximum values from GTSM output. The analysis focuses on selected GTSM points shown in Fig. 2, covering the IPCC reference regions of Northern Europe (NEU), Russian Far East (RFE), Northeast North America (NEN), and East Asia (EAS). These regions were chosen not only for their diverse climatic conditions but also because they experience frequent and intense storm surge activity. Coastal areas in these regions are particularly vulnerable to extreme sea levels due to the combined effects of strong winds, shallow coastal bathymetry, and storm tracks that favor surge generation. For each location, the annual maximum surge height and its corresponding date were identified, ensuring consistency in event-location correspondence. On these dates, daily maximum values of key forcing parameters–Charnock parameter, wind speed difference, and air density–were extracted from the model inputs.

The first column of Fig. 9 ($\text {GTSM}_{\text {CAD}} - \text {GTSM}_{\text {IFS}}$) examines the role of air density differences in surge generation. In NEU (Fig. 9a), RFE (Fig. 9d), NEN (Fig. 9g), and EAS (Fig. 9j), surge differences are generally small, ranging between -0.2 and +0.1 m, with minimal variability. This suggests that while air density influences momentum transfer, its overall impact on storm surge remains moderate. However, higher surge heights tend to be more affected by air density variations, as stronger storms associated can enhance wind stress and consequently, storm surge generation. The second column ($\text {GTSM}_{\text {NST}} - \text {GTSM}_{\text {CAD}}$) highlights the influence of atmospheric boundary layer stability on surge height. Across all regions (Fig. 9b, Fig. 9e, Fig. 9h, and Fig. 9k), surge differences stay within a narrow range, typically around ±0.1 m. This suggests that atmospheric stability has little effect on surge height. The third column ($\text {GTSM}_{\text {FIX}} - \text {GTSM}_{\text {NST}}$) highlights the impact of Charnock parameter differences on surge height. Surge height differences range from -0.2 to +0.6 m in NEU (Fig. 9c), -0.6 to +0.4 m in RFE (Fig. 9f), -0.8 to +0.6 m in NEN (Fig. 9i), and -0.3 to +0.3 m in EAS (Fig. 9l). The impact of the variable Charnock parameter is most pronounced in NEU and NEN, where surge height differences reach up to 0.6 m and 0.8 m, respectively. However, these differences are not always directly linked to local Charnock values but may depend on conditions in surrounding areas or preceding the peak. This suggests that interactions between local and regional wind stress patterns, as well as temporal variability in surface roughness, can play an important role. Overall, Fig. 9 shows the varying sensitivity of storm surge to different atmospheric and surface roughness parameters. While air density and atmospheric stability generally produce small surge variations, the Charnock parameter exhibits a much stronger influence.

Figure 10 compares modeled annual maximum surge heights against observations for 300 stations over a 10-year period, representing approximately 3000 data points per plot. The top row (Fig. 10a–d) evaluates four experiments: $\text {GTSM}_{\text {IFS}}$, $\text {GTSM}_{\text {CAD}}$, $\text {GTSM}_{\text {NST}}$, and $\text {GTSM}_{\text {FIX}}$. The experiment ($\text {GTSM}_{\text {IFS}}$) has a slope of 0.88 and an RMSE of 0.23 m, indicating a general underestimation of observed surges by approximately 12% (Fig. 10a). Simplifying the variable air density in $\text {GTSM}_{\text {CAD}}$ experiment results in a slope of 0.87, leading to an additional 1% underestimation in surge heights, with a minimal change in RMSE (Fig. 10b). Further neglecting atmospheric stability effects in $\text {GTSM}_{\text {NST}}$ reduces the slope to 0.86, again increasing the underestimation by another 1%, but the impact on RMSE is negligible (Fig. 10c). The largest impact comes from switching from a variable to a constant Charnock parameter in GTSM$_{\text {FIX}}$, which lowers the slope to 0.82, introducing an additional 4% underestimation in modeled surges (Fig. 10d). Together, these simplifications increase the overall underestimation of annual maximum surges from 12% in GTSM$_{\text {IFS}}$ to 18% in GTSM$_{\text {FIX}}$. The bottom row (Fig. 10e–g) examines model differences relative to observations by computing residuals. The differences between GTSM$_{\text {CAD}}$ and GTSM$_{\text {IFS}}$ (Fig. 10e) and between GTSM$_{\text {NST}}$ and GTSM$_{\text {CAD}}$ (Fig. 10f) are relatively small, with each simplification contributing a gradual 1% decrease in surges. However, the transition from GTSM$_{\text {NST}}$ to GTSM$_{\text {FIX}}$ results in the most pronounced change (Fig. 10g), leading to an average 4% decrease in surge heights, with even greater reductions at higher observed surges, although the correlation remains relatively low (-0.48). These findings highlight that while simplifications in air density and stability have minor effects, the choice of the Charnock parameter has the most significant impact on surge height predictions.

## Discussion and conclusion

This study investigates the sensitivity of surge height predictions to variations in air density, atmospheric stability, and Charnock parameter. The results show the importance of dynamically adaptive drag formulations in enhancing GTSM performance. Case studies of Storm Xaver (2013) and Hurricane Fiona (2022) demonstrate that the most comprehensive parameterization, $\text {GTSM}_{\text {IFS}}$, provides the best overall performance, while simplified approaches ($\text {GTSM}_{\text {CAD}}$, $\text {GTSM}_{\text {NST}}$, and $\text {GTSM}_{\text {FIX}}$) introduce increasing biases. Notably, the variable Charnock parameterization ($\text {GTSM}_{\text {NST}}$) had the largest impact on reproducing surge dynamics. The most advanced experiment, $\text {GTSM}_{\text {IFS}}$, incorporates stress-equivalent winds, variations in air density, and a wave-state-dependent Charnock parameter. By adjusting for temperature, pressure, and humidity variations, it improves air-sea interaction representation, leading to the best agreement with observed surges. For Storm Xaver, $\text {GTSM}_{\text {IFS}}$ achieves the lowest RMSE at 0.14 m. A simplification of this approach, $\text {GTSM}_{\text {CAD}}$, assumes a constant air density while retaining neutral wind corrections and a dynamically varying Charnock parameter. This reduces computational complexity while still accounting for surface roughness variability. The results indicate that it maintains much of the accuracy of $\text {GTSM}_{\text {IFS}}$, with an RMSE of 0.15 m. The slight increase in error suggests that air density variations have a relatively minor impact on storm surge predictions. Further simplifying, $\text {GTSM}_{\text {NST}}$ eliminates explicit stability corrections and instead uses actual 10-m winds rather than neutral winds. It retains a wave-state-dependent Charnock parameter but does not explicitly correct for atmospheric stability effects. This leads to a slight increase in RMSE to 0.16 m, indicating that while stability adjustments contribute to accuracy, variable roughness remains the dominant factor in reducing bias. The simplest experiment, $\text {GTSM}_{\text {FIX}}$, applies a fixed Charnock parameter and assumes constant air density, providing the least dynamic representation of wind stress. This leads to the largest underestimation of storm surges, particularly during extreme events. For Storm Xaver, RMSE increases to 0.21 m, illustrating the limitations of a constant-drag formulation. For Hurricane Fiona, the results show a similar tendency to those of Storm Xaver, but the differences between experiments are less pronounced according to tide gauge data. While the $\text {GTSM}_{\text {FIX}}$ experiment estimates peak surge heights at around 1.8 m, the $\text {GTSM}_{\text {NST}}$ experiment increases surge predictions by up to 0.30 m, aligning with unofficial reports of surges exceeds 2.1 m above ground level (Pasch et al. 2023). However, improvements from surface roughness, air density, and stability adjustments are less evident in the tide gauge comparison in case of Hurricane Fiona, likely due to the gauges’ limited coverage relative to the areas of maximum surge. These results show the key role of variable Charnock in capturing extreme surge events, compared to the relatively minor improvements provided by adjustments to air density and stability corrections. Overall, as the complexity of the parameterization decreases, surge height predictions become less accurate. While the inclusion of air density variations and stability corrections provides incremental improvements, the dominant factor influencing model accuracy is the use of a variable Charnock parameter. These results are consistent with previous studies on sea-state effects, including Charnock parameterization and wave age dependence (Mastenbroek et al. 1993; Bertin et al. 2015; Muller et al. 2014; Pineau-Guillou et al. 2020). However, further case studies are required to strengthen these conclusions.

When it comes to regional surge heights, the results demonstrate that while variable Charnock has the largest impact on height differences, the combined effects of atmospheric boundary layer stability and air density adjustments also can influence regional variability for high surge heights. Additionally, a comparison of global annual maximum surge values from various GTSM experiments with observations shows the benefits of parameterization adjustments. Collectively, modifications to surface roughness, atmospheric stability, and air density reduced the model’s overall bias, lowering underestimation from 18% ($\text {GTSM}_{\text {FIX}}$) to 12% ($\text {GTSM}_{\text {IFS}}$). Of these, the variable Charnock parameter contributed a 5% improvement, while stability and air density adjustments each provided a further 1% reduction. The results demonstrate that parameterization adjustments address underestimation issues, enhancing the model’s overall accuracy. Additionally, by employing these parameterizations, reliance on constants specifically calibrated for extreme surge events is reduced, potentially improving water level forecasts under less extreme conditions. These findings align with earlier studies, such as Muller et al. (2014), which showed that variable Charnock parameters helped reduce peak surge errors along the French coast. Similarly, Harter et al. (2024) identified persistent underestimation of extreme surges in statistical reconstructions and highlighted the importance of using wind stress and wave height predictors to reduce biases in extreme surge modeling.

This study emphasizes the role of sea surface drag formulations, particularly the variable Charnock parameter, in improving storm surge modeling. Among the tested parameterizations, $\text {GTSM}_{\text {IFS}}$ provides the most physically realistic representation, but results show that the dominant improvement comes from variable surface roughness, while air density and stability corrections offer only minor refinements. The comparison of different models demonstrates that reducing complexity increases bias, with the most significant underestimation occurring in $\text {GTSM}_{\text {FIX}}$, which assumes a fixed Charnock parameter. Given that air density variations and atmospheric stability correction have a negligible effect on surge heights, this study questions their necessity in operational models and emphasizes the need to prioritize dynamic sea-state-dependent drag formulations. This study highlights that variable Charnock is essential for accurate storm surge modeling, while air density and stability adjustments remain secondary considerations.

Despite the presented results, several limitations with the performance of global surge models remain and could be addressed in future work. ERA5 reanalysis data, while widely used, tends to underestimate high wind speeds, as documented in previous studies (Dullaart et al. 2020; Xiong et al. 2022; Gandoin and Garza 2024; Zhang et al. 2024; Pineau-Guillou et al. 2018). This underestimation contributes to persistent biases, especially during storms, emphasizing the need for higher-resolution or corrected datasets or bias-correction methods such as using machine learning (Tedesco et al. 2024; Giaremis et al. 2024; Zampato et al. 2006). For instance, emerging machine learning techniques have effectively improved storm tide forecasting by correcting systemic biases, as demonstrated during Hurricane Ian, with notable accuracy gains and low computational costs (Giaremis et al. 2024). Our results show that model validation is particularly challenging in regions lacking long-term observational records, such as parts of Africa, the southern Pacific, and polar areas. While the use of satellites is promising (Andersen et al. 2015), their coverage also near the poles remains poor.

The findings of this study have implications for both operational forecasting and climate research. In operational settings, incorporating these parameterization adjustments into GTSM could improve extreme event forecasts, aiding disaster preparedness and risk management in coastal regions, though computational efficiency remains a key consideration. For climate studies, enhanced drag formulations may better capture storm surge dynamics under changing climate conditions. However, it is important to note that CMIP simulations, including higher-resolution experiments such as HighResMIP, do not provide the necessary surface-layer parameters such as detailed wind stress and sea level pressure fields at fine scales to directly apply these formulations, which limits their immediate use in such models (Muis et al. 2023). One way to address this gap is through regional downscaling or by embedding high-resolution models within broader global frameworks, which can help connect the coarse outputs of global models with the finer detail required for coastal impact studies (Chaigneau et al. 2022). In addition, recent progress in machine learning offers possibilities for simulating the smaller-scale processes that global models miss, without intensive computational resources (Yuval and O’Gorman 2020). Bias-corrected parameterizations could help address underestimations in reanalysis data and global climate models, improving future surge risk assessments. Additionally, integrating these refinements into coupled climate-ocean models would provide deeper insights into the interactions between storm intensity and wind stress.

Future work should focus on refining wave-atmosphere coupling and reducing reliance on empirically tuned constants to improve model accuracy under diverse conditions. Advancements in high-resolution meteorological forcing and better observational datasets will be key to further enhancing storm surge predictions. Additionally, improving performance in the Arctic region presents specific challenges, including the limited coverage of observational networks and the need to account for sea-ice dynamics, which are essential for improving regional forecast accuracy. Given the minor impact of air density and stability corrections, our results show the need to focus on dynamic sea-state-dependent drag formulations as the key driver of improved surge predictions in operational models.

## Data Availability

Water level observations used in this study were obtained from the GESLA-3 database (https://www.gesla.org/) and the Canadian tides and water levels data archive (https://tides.gc.ca/en). Both datasets are publicly available and were used for model validation. The data generated and analyzed in this study are publicly available on 4TU.ResearchData at https://doi.org/10.4121/923c1f55-01f7-4140-b9ce-ab3be5a617c7.

## Appendix Air density correction for winds

The formula for wind stress (Eq. 1) shows a direct proportionality to air density. Using the default settings of GTSM, which have been used in previous studies (Dullaart et al. 2020; Muis et al. 2016, 2019, 2020), air density is assigned a constant value of 1.20 kg/m$^3$. However, an air density of 1.20 kg/m$^3$ corresponds to conditions at sea level with a temperature of $21^\circ$C and air pressure of 1013.25 hPa. In reality, air density shows variability in space and time depending on air pressure, temperature and humidity.

Although air density over the oceans is available in ERA5 dataset at a horizontal resolution of $0.5^\circ \times 0.5^\circ$, its resolution is lower than that of other model parameters because it is derived from the wave model. To address this and ensure consistency with other forcing parameters, air density ($\rho _a$) is dynamically calculated using air temperature, dew point temperature, and air pressure from ERA5 dataset (see Table 1). Stress-equivalent winds are also computed from neutral winds through an iterative correction process that involves updated calculations of air density and drag coefficients. Following the methodology described by de Kloe et al. (2017), the steps to compute stress-equivalent winds are as follows:

1. Compute water vapor pressure based on dew point temperature:
  A1 $$\begin{aligned} e = e_0 \cdot \exp \left( 17.502 \cdot (T_d - T_0) \cdot (T_d - 32.19)\right) \end{aligned}$$
  where $e_0$ is the water vapor saturation pressure over water (611.21 Pa) at the triple point temperature $T_0$.
2. Compute specific humidity based on water vapor pressure and air pressure:
  A2 $$\begin{aligned} q_v = \frac{e}{p + \epsilon ^* \cdot (p - e)} \end{aligned}$$
  where $\epsilon ^* = \frac{R_v}{R_d} - 1$, with $R_v$ and $R_d$ the gas constants for water vapor (461.5249 J kg$^{-1}$  K$^{-1}$ ) and dry air (287.0596 J kg$^{-1}$  K$^{-1}$), respectively.
3. Compute virtual temperature based on air temperature and specific humidity:
  A3 $$\begin{aligned} T_v = T \cdot (1 + \epsilon ^* \cdot q_v) \end{aligned}$$
4. Compute air density based on air pressure and virtual temperature:
  A4 $$\begin{aligned} \rho _a = \frac{p}{R_d \cdot T_v} \end{aligned}$$
5. Compute the drag coefficient iteratively using Charnock and wind speed, applying logarithmic wind profile theory:
  A5 $$\begin{aligned} u_*&= \frac{\mathcal {K} \cdot U_{10}}{\ln \left( \frac{10}{z_0}\right) },&z_0&= \alpha _{\text {Ch}} \frac{u_*^2}{g},&C_D&= \left( \frac{u_*}{U_{10}}\right) ^2 \end{aligned}$$
6. Compute the stress-equivalent friction velocity:
  A6 $$\begin{aligned} u_{*s} = u_* \sqrt{\frac{\rho _a}{\rho _{GTSM}}} \end{aligned}$$
7. Compute the roughness length $z_0$ using stress-equivalent friction velocity and the Charnock parameter from ECMWF ($\alpha _{\text {Ch}}$) and gravitational acceleration ($g = 9.813~\text {m/s}^2$):
  A7 $$\begin{aligned} z_0 = \alpha _{\text {Ch}} \frac{u_{*s}^2}{g} \end{aligned}$$
8. Compute the stress-equivalent wind $U_{10S}$ based on the stress-equivalent friction velocity $u_{*s}^2$ and the roughness length $z_0$, assuming a logarithmic profile:
  A8 $$\begin{aligned} U_{10S} = \frac{\log \left( \frac{10}{z_0}\right) }{\mathcal {K}} \cdot u_{*s} \end{aligned}$$
